# Use of measures and measurement-based care in child and youth mental health: a survey of Australian practitioners

**DOI:** 10.1080/00049530.2024.2426662

**Published:** 2024-11-18

**Authors:** Lucy A. Tully, Janice Kan, Adrienne Turnell, Rebecca McLean, Trisha Nowland, Olivia Liew, Lindsay McFarlane, David J. Hawes, Mark R. Dadds

**Affiliations:** School of Psychology, University of Sydney, Sydney, Australia

**Keywords:** Measurement-based care, child/youth mental health, evidence-based practice

## Abstract

**Objective:**

Use of measures by practitioners in mental health (MH) is a cornerstone of evidence-based practice and essential to high-quality service provision. Session-by-session measure use, known as Measurement-Based Care (MBC), has been shown to improve treatment engagement and outcomes, yet little is known about the use of measures or MBC in Australian child and youth MH practitioners. This study surveyed Australian child and youth MH practitioners to examine the frequency of measure use, what outcomes are measured, and what facilitates measure use.

**Method:**

This survey included Australian practitioners (*N* = 205) working in child and youth MH.

**Results:**

Most practitioners reported using measures at some stage during treatment, but around 1 in 7 did not use measures at all. Only 10% used measures for every session or most sessions, which is characteristic of MBC. Symptom severity was measured by 84.3% of practitioners but only 35.6% measured goal attainment and 16.7% therapeutic alliance. The top facilitators of measure use endorsed by practitioners included free measures, better platforms to administer measures, and briefer measures.

**Conclusions:**

There is room for improvement in the use of outcome measures by Australian child and youth MH practitioners, and specifically in the use of MBC, which may improve client engagement and outcomes. Implications for the implementation of MBC are discussed.

## KEY POINTS

**What is already known about this topic:**

1. Use of measures by practitioners is an important part of evidence-based practice.
2. Use of Measurement-Based Care can improve engagement and outcomes.
3. There is a lack of research about the use of measures and MBC by Australian child and youth practitioners.

**What this topic adds:**

1. About one in seven Australian children and youth practitioners do not use measures at all.
2. Very few Australian practitioners use measures every session, which is characteristic of MBC.
3. This study identified several facilitators that are easy to implement and can increase measure use by practitioners.

The collection of clinical data through valid and reliable measurement is a cornerstone of evidence-based practice and is essential to evidence-based assessment in mental health (MH) (Hunsley & Mash, 2007). Evidence-based assessment includes collecting data about a range of constructs, such as diagnoses, symptom severity, impact, goal attainment, and therapeutic alliance. Evidence-based assessment can guide case conceptualisation and treatment planning, as well as inform the evaluation of outcomes, discharge planning, and service evaluation (Youngstrom et al., 2017). In recent years, there has been growing interest in the practice of Measurement-Based Care (MBC), whereby regular (e.g., session-by-session) assessment is conducted to monitor client progress, facilitate collaborative decision-making, reduce drop out, and improve outcomes from MH interventions. There is considerable evidence supporting the efficacy of MBC in interventions for adult MH (Lambert et al., 2018), and emerging evidence in youth MH (Tam & Ronan, 2017). Despite the importance of evidence-based assessment, there is little evidence about the routine use of measures in practice, especially in child and youth MH (CYMH) in Australia. Such evidence would inform strategies to increase the use of evidence-based assessment and improve MH service quality. This paper describes the findings of a survey with Australian CYMH practitioners examining the use of measures, perceptions of facilitators of measure use, and predictors of measure use.

Research supporting the use of evidence-based assessment for improving client outcomes from interventions has primarily focused on MBC in adult MH. Among adult MH interventions, MBC has been associated with improved symptom severity, reduced drop out and deterioration, shortened length of treatment (e.g., Aboraya et al., 2018; Fortney et al., 2017; Lambert et al., 2018; Rognstad et al., 2023) and reduced cost of care (Delgadillo et al., 2021). In relation to youth, recent meta-analytic reviews have found that the use of MBC improved symptom severity, functioning and goal attainment for interventions for young people aged 10 to 19 years, with small to medium effect sizes (Rognstad et al., 2023; Tam & Ronan, 2017). Based on existing evidence, the APA Presidential Task Force on Evidence-Based Practice (2006) has recommended MBC as integral to evidence-based practice particularly within adult MH.

Despite the beneficial effects of MBC, emerging evidence suggests that measures are not routinely used to inform clinical practice. For example, a survey in the US with 504 MH practitioners found only 5.2% used standardised progress measures every one to two sessions, while 61.5% did not use them consistently (Jensen-Doss et al., 2018). Similarly, a survey of 1668 Canadian psychologists found only 12% used measures routinely (Ionita & Fitzpatrick, 2014). For practitioners who work exclusively in CYMH, Cho et al. (2021) conducted two large-scale multidisciplinary surveys in the US (*N* = 2,575) and found most practitioners reported using measures in pre-treatment assessments (93%, 95%) “often, all or most of the time”. Fewer reported using measures during treatments such as weekly or monthly (77% across both surveys), and at the end of treatment (68%, 73%). Additionally, around half reported using measures “often, all or most of the time” throughout the entire treatment process (49%, 55%). A survey of 186 UK practitioners in CYMH services found that while 65% of clinicians reported using measures throughout treatment, only 29% did so systematically (Johnston & Gowers, 2005). While it is difficult to compare the findings across studies due to differences in methodology and definitions of what constitutes “routine” measure use, it appears that routine measure use throughout treatment varies widely from 12% to 55%. The differences are potentially due to a number of factors including the differing health contexts in different countries, such as national initiatives promoting use of routine outcome measures in some health systems (e.g., UK IAPT model; Wakefield et al., 2021); the varying resources available for MBC across countries; and, the timing of these surveys being conducted several years apart in a context where there has been greater MBC use in the past 5–10 years. However, the largely low rates of frequent use of measures in previous surveys is concerning given that routine use of measures is essential to enable clinicians to identify client deterioration or non-response in a timely manner, which may be missed with less frequent use of measures (De Jong et al., 2023).

Despite several previous studies examining the frequency of measure use by practitioners, a survey of Australian CYMH practitioners has yet to be conducted. As mentioned, the rates of measure use are likely to vary according to specific healthcare contexts, and findings from previous surveys cannot be generalised to the Australian context. Conducting a survey of Australian CYMH practitioners is important since prevalence rates of childhood MH problems in Australia are high and not decreasing (Sawyer et al., 2018). While there are a number of reasons for these stable prevalence rates, strategies are needed to improve the effectiveness of interventions, especially since evidence suggests the effect sizes for interventions for CYMH have not increased over time, and in some instances, are decreasing (Weisz et al., 2019). Should low rates of routine measure use be found, strategies to increase the use of measures may provide a novel and cost-effective method for improving the effectiveness of CYMH interventions, potentially impacting on the prevalence of mental health problems in Australian children.

To date, there is limited research exploring which outcomes are measured routinely. Symptom severity and functioning have been the most frequently measured outcomes in MBC to determine client progress. However, studies have recently started to measure other constructs known to influence engagement and outcomes, including quality of life, readiness for change, goal attainment, and therapeutic alliance (e.g., Kazdin & McWhinney, 2018; Schmidt et al., 2014), as well as using individualised, person-centred measures that may be more clinically meaningful than objective measures (Lloyd et al., 2019). This move beyond symptom severity is supported by research which found larger effect sizes for MBC that included measures of quality-of-life, functioning and therapeutic alliance than MBC that included only MH symptoms (Rognstad et al., 2023). The only practitioner survey to examine the type of outcomes measured found the majority reported assessing behaviour and symptoms (93.2%) and general functioning (82.6%), with fewer assessing social functioning (59%; Johnston & Gowers, 2005). Notably, this study did not ask whether other outcomes were assessed, such as the therapeutic relationship or goal attainment. Further research about types of measures is needed to identify practice gaps and inform training in evidence-based assessment.

Previous research focused on identifying barriers to routine measure use has identified a range of interrelated barriers. These include the time-consuming nature of administration, client burden, lack of training, lack of practical assessment tools, low completion rates, and concerns about reliability, validity and effectiveness of measures (Batty et al., 2013, Cho et al., 2016; Gleacher et al., 2016, Hatfield & Ogles, 2004; Ionita et al., 2020; Johnston & Gowers, 2005; Kotte et al., 2016). There has been much less research on practitioners’ perceptions of facilitators of routine measure use. Facilitators of measure use are important to explore as they may inform new initiatives to increase the routine use of measures. In one such study, Ionita et al. (2020) presented participants with a list of facilitators to overcome key barriers and identified that facilitators such as “discovering measures take less than 5 or 10 minutes to complete” and “hearing positive testimonial” were rated highest to address the number one barrier of “requiring too much time”. Further research on facilitators of measure use is needed to identify features to integrate into evidence-based assessment training (Cho et al., 2021), and to identify facilitators specific to CYMH.

Studies have also examined factors that may predict measure use. At the practitioner level, research has found years of professional experience was related to the use of measures, with more years reducing the likelihood of monitoring progress at all, although years of experience was unrelated to the frequency of measure use (Jensen-Doss et al., 2018). Research has also found that psychologists are more likely to use measures than professionals from other disciplines (Ionita & Fitzpatrick, 2014), although not all research has supported this finding (Jensen-Doss et al., 2018). At the service level, research has found that the likelihood of using any measures at all, and the frequency of use of measures, was lower for practitioners working in private practice as compared with other service contexts (Jensen-Doss et al., 2018). Thus, fewer years of experience, working in service contexts other than private practice, and working in the psychology profession may be associated with increased use of measures. However, since these studies have not specifically focussed on practitioners working in CYMH, it is important to examine whether these factors are associated with measure use in this context.

In summary, evidence suggests infrequent use of measures by practitioners, especially routine measure use that is integral to MBC, although this research has not been specific to CYMH. There is also little research on the type of outcome measures used, as well as practitioners’ perspectives about facilitators of measure use. Thus, there is currently little knowledge to draw from regarding strategies that may support Australian MH practitioners to increase their use of measures in clinical practice. A survey is needed to obtain a benchmark and to identify appropriate strategies to increase evidence-based assessment. To address these issues, the aim of the current study was to survey Australian CYMH practitioners to examine the frequency of measure use, outcomes measured, and facilitators for measure use. The current study also examined whether practitioners endorsed different facilitators based on previous use or non-use of MBC and explored factors that predicted the frequency of measure use. Three predictors were examined: discipline, years of experience, and practice context. Based on previous research, it was expected that fewer years of clinical experience and not working in private practice would be associated with greater measure use (Hypothesis 1). No hypotheses were formed for psychology discipline due to mixed findings from previous research.

## Method

### Procedure

The Human Research Ethics Committee at the University of Sydney provided ethics approval for the study. The survey, which took approximately 20 minutes to complete, was anonymous. Participants completing the survey could choose to enter a draw to win a gift voucher.

### Participants and recruitment

Recruitment involved outreach to a range of government and non-government child and family services around Australia, primarily via social media posts and distribution of flyers. In addition, the survey was promoted via professional bodies and networks.

Advertisements directed interested participants to the online survey hosted on REDCap between August 2022 and March 2023. Potential participants were told that the survey was about the perspectives and experiences of Australian practitioners working in child and youth mental health. Along with questions about measure use, the survey included questions about waitlist times, evidence-based practice, and training needs, which are reported elsewhere (Kan et al., under review).

### Measures

Questions included in the survey were selected through a literature review and input from a team of psychologists and researchers with expertise in CYMH. The draft survey was pilot tested with a small convenience sample of practitioners, and based on feedback, items were revised to improve clarity before being included in the final survey.

Information was collected on practitioner’s gender, profession, location, highest level of education, years of experience working in their profession and in CYMH. Practitioners were asked if they used outcome measures in their practice (*yes* or *no*). For those who endorsed using measures, they were asked to indicate when they used outcomes measures (i.e., *assessment/initial appointment*, *throughout treatment*, or *end of treatment/discharge*) and how often they used measures (i.e., *every session*, *most sessions*, *at specific time points*, *occasionally*, and *when I remember*). Responses to these questions were recorded in order to create a composite “MBC use” and “MBC non-use” variable for further analyses. Respondents who endorsed that they did not use measures at all, did not use measures throughout treatment, used measures during treatment occasionally or only when they remembered were coded as MBC non-use. Those who endorsed using measures throughout treatment with a frequency of every session, most sessions, or at specific time points were coded as MBC use. We included the use of measures at specific time points in addition to using measures most and every session as part of “MBC use”, given this suggests an intentional use of measures by practitioners as part of treatment planning.

Respondents were asked which outcomes they measured (i.e., *diagnosis, symptom severity, impact of illness on functioning, goal attainment, therapeutic alliance, session feedback, service feedback*) and what facilitators would increase their use of measures, including: *measures being available in the language my clients speak, more valid measures for the population I work with, shorter measures, better tools to administer measures (e.g., easy to use software), free/no cost measures, more time to administer measures, more training on how to use measures, and more workplace support*. The total number of facilitators endorsed was calculated by computing a total for each facilitator endorsed, which ranged from 0 to 8.

### Analysis plan

Variables were analysed using frequencies and descriptives with SPSS version 26. To examine differences between MBC use versus non-use groups, a series of chi-square tests were conducted for facilitators endorsed, and a t-test was performed for the total number of facilitators. A binary logistic regression analysis was conducted to examine predictors of MBC use, with one continuous predictor variable (years of experience) and two categorical variables (profession and workplace setting). The latter two variables were recoded for use in the logistic regression, with profession being recoded as “psychologist” or “other” profession, and workplace setting recoded as “private practice” or “other” setting.

## Results

### Practitioner characteristics

Of the 217 practitioners who began the survey, 205 (94.5%) completed the questions about measures. The demographic and professional characteristics of respondents are reported in Table 1. Most respondents were female (87.3%) and had post-graduate level of education (64.4%). Two-thirds lived in metropolitan areas in Australia, with the majority residing in New South Wales (61.5%). Psychologists (70.7%) were the most common respondents, followed by social workers (11.5%). In terms of practice setting, just under half of the sample indicated that they worked in a private practice setting (48.3%) and a similar proportion in public health (49.3%). Respondents were on average 43.1 years of age (*SD* = 11.8; range 23 to 79 years) and had an average of 13.8 years working in their profession (*SD* = 10.9; range = 1 to 56 years), and 13.4 years working in CYMH (*SD* = 10.7; range = 1 to 48 years).

**Table 1.** Demographic and professional characteristics of the sample.

Characteristic	n	%
Gender		
Female	179	87.3
Male	25	12.2
Non-binary	1	0.5
Highest level of education		
Undergraduate university degree	26	12.7
Postgraduate university degree	132	64.4
Doctorate	47	22.9
Location		
Metropolitan	139	67.8
Regional	66	32.2
State or territory		
New South Wales	126	61.5
Queensland	32	15.6
Victoria	24	11.7
Western Australia	18	8.8
South Australia	2	1.0
Northern Territory	2	1.0
Australian Capital Territory	1	0.5
Profession^a^		
Psychologist (Clinical/General/Provisional)	145	70.7
Social worker	22	10.7
Nurse	17	8.3
Counsellor	4	1.9
Psychiatrist	4	1.8
Case worker	2	1.5
Occupational therapist	1	0.5
School counsellor	1	0.5
Other^b^	9	7.8
Practice setting/location^a^		
Private practice	99	48.3
Public health	101	49.3
School	26	12.7
University	19	9.3
Non-government organisation	8	3.9
Other^c^	7	3.4

^a^Denotes items where participants could choose more than one response; ^b^Other professions include: Art therapist; mental health dietician; peer support worker; physiotherapist; exercise physiologist; specialist school principal; ^c^Other practice setting/location include: client’s residence, out of home care.

### Use of measures

The majority of respondents (*n* = 172, 83.9%) indicated they used outcomes measures in their practice at least occasionally, with only 16.1% (*n* = 33) indicating they did not use outcome measures at all. Of those who used outcome measures, 87.8% (*n* = 151) reported that they used measures at assessment, 78.5% (*n* = 135) reported that they used measures throughout treatment, and 80.2% (*n* = 138) reported that they used measures at the end of treatment. For the entire sample, this equates to 73.7% using measures at assessment, 65.9% using measures throughout treatment and 67.3% using measures at discharge.

Of those who used measures throughout treatment, the frequency of measure use is displayed in Table 2. Of the sample, 59.3% indicated they used measures at specific time points during treatment, 18.3% used them occasionally, and only a small proportion used them every session (5.9%) or most sessions (9.6%). Based on the entire sample of 205 practitioners, this equates to only 10.2% of the respondents using measures most or every session.

**Table 2.** Frequency of use of outcome measures during treatment for those endorsing measure use.

Timepoint	n	%
At specific time points (e.g., Session 3, 5, 10 etc)	80	59.3
Occasionally	25	18.5
Most sessions (but not every session)	13	9.6
When I remember	9	6.7
Every session	8	5.9

*N* = 135.

The most frequent type of outcome measure that practitioners endorsed using was symptom severity (*n* = 145, 84.3%). Just over half endorsed measuring diagnosis (*n* = 97, 56.4%) or impact on functioning (*n* = 97, 56.4%). Just over one-third endorsed measuring goal attainment (*n* = 62; 35.6%), and just over a quarter measured service feedback (*n* = 49, 28.4%) or session feedback (*n* = 48, 27.9%). Finally, only 16.7% (*n* = 29) endorsed measuring therapeutic alliance.

Of the sample, 101 (49.3%) respondents were categorised in MBC use and 104 (50.7%) were categorised as MBC non-use. Table 3 lists the number of facilitating factors that practitioners endorsed for the overall sample, and separately for the MBC use and non-use groups. For the entire sample, nearly half of respondents (48.8%) endorsed free measures, followed by better tools to administer measures (45.4%). Shorter measures and more time to administer measures were endorsed by 31.2% and 30.2%, respectively, and 27.3% endorsed more valid measures for specific populations. More training in use of measures was endorsed by 19.5%, and more workplace support, and measures being available in client languages was endorsed by 14.1% and 13.7% respectively. Those in the MBC use group endorsed significantly more facilitators overall than those in the MBC non-use group (2.7 vs 1.9), with a significantly greater proportion endorsing four facilitators: free measures, better tools to administer measures, shorter measures, and more valid measures for target population.

**Table 3.** Facilitators endorsed for increasing use of measures for the entire sample and for MBC use and MBC non-use groups.

	Overall sample (n = 205)		MBC use		MBC non-use		Chi square
Facilitator endorsed	n	%	n	%	n	%	
Free measures	100	48.8	62	61.4	38	36.5	12.66***
Better tools to administer measures	93	45.4	58	57.4	35	33.7	11.68***
Shorter measures	64	31.2	42	41.6	22	21.2	9.96**
More time to administer measures	62	30.2	32	31.7	30	28.8	0.19
More valid measures for the population I work with	57	27.3	35	34.7	21	20.2	5.39*
More training on how to use measures	40	19.5	17	16.8	23	22.1	0.91
More workplace support	29	14.1	18	17.8	11	10.6	2.21
Measures available in the language my clients speak	28	13.7	13	12.9	15	14.4	0.11
	M	SD	M	SD	M	SD	T-test
Mean number of facilitators endorsed	2.30	1.78	2.74	1.59	1.88	1.86	−3.58***

Multiple responses permitted, so percentages do not add up to 100%. *N* = 205.

*p < .05, **p < .01, ***p < .001.

A logistic regression to examine factors predicting MBC use or non-use found that the model overall was not significant [χ^2^ (3, *N* = 205) = 4.05, *p* = 0.26]. This indicates that the model was not able to distinguish between MBC users and non-users based on the three predictor variables.

## Discussion

This study surveyed Australian CYMH practitioners to explore the frequency of measure use, outcomes measured, and facilitators for measure use. The survey found that measures were used by the majority of practitioners, but around one in seven practitioners (16.1%) did not use measures at all in their clinical practice, which is concerning given that evidence-based assessment is essential to high-quality service provision. Furthermore, across the entire sample, only 10% used measures every session or most sessions, indicating that use of session-by-session measures was low overall.

For the entire sample, almost three-quarters of practitioners reported using measures at assessment and two-thirds used them at discharge and throughout treatment. The high rates of measure use at assessment, discharge, and at specific time points throughout treatment may be reflective of the Australian MH landscape. That is, specialised public sector practitioners are mandated to collect routine outcome data at admission, discharge, and, for longer episodes of MH care, at designated review points (Burgess et al., 2015). Relatedly, under the Medicare Benefits Schedule, private MH practitioners seeking government rebated services for their patients are required to provide a written report to referring practitioners about assessments carried out, treatment provided, and recommendations for further management. Given these outcome reporting requirements, it is perhaps unsurprising that our sample of practitioners are endorsing measure completion at such high rates. However, when asked to endorse the frequency of measure use, only a minority of practitioners reported using measures regularly. The proportion of practitioners using measures “every” or “most” sessions in this survey (10%) was lower than has been found in previous surveys in CYMH (Cho et al., 2021; Johnston & Gowers, 2005), although it should be noted that different methodologies make comparison of findings difficult. This finding suggests that MBC is rarely used by CYMH practitioners in Australia, despite the potential benefits for improving client engagement and outcomes. As ongoing use of measures is not mandated for MH services in Australia, it might be that practitioners’ motivation for measure use is driven by external reporting requirements rather than the value of MBC to improve MH outcomes. It is important to note however, that this survey only asked practitioners about the frequency of measure use and did not ask about the provision of feedback and collaborative discussion with clients to inform treatment planning, which is also an integral part of MBC.

In terms of outcomes measured, the most frequent construct measured was symptom severity, followed by diagnosis and functioning. This is perhaps unsurprising and may reflect the function of outcome measures used by this sample. Given practitioners predominantly endorsed measure use at assessment and discharge, this might reflect the need for measures to determine severity of presentation and treatment outcomes, especially considering previously mentioned reporting requirements. Only 16.1% of practitioners reported measuring therapeutic alliance. Given that research has demonstrated that the therapeutic relationship is highly predictive of client outcomes, further emphasis on the need for monitoring this construct throughout treatment is needed (e.g., Kazdin & McWhinny, 2018). Routine monitoring of the therapeutic alliance enables ruptures to be identified early and action to be taken to repair the relationship, potentially preventing drop out or poor treatment response. Person-centred measures such as measures of goal attainment were also infrequently used, despite these measures potentially being more clinically meaningful than objective measures (Lloyd et al., 2019). Selection of measures will vary depending on the intervention and the outcomes targeted, and it is important for participant burden to be considered when using multiple measures. It should be noted that there is little research to guide practitioners about what constructs are best to measure as part of MBC, and this is a direction for future research.

In terms of facilitators for measure use, the top facilitator endorsed by almost half the sample was free measures. There is a paucity of previous research on facilitators to compare current findings, although research on barriers points to cost as a key factor (e.g., Hatfield & Ogles, 2004). Almost half of the respondents in the present survey also endorsed having better tools or platforms to administer measures, supporting the limited research in MBC conducted elsewhere (Murphy et al., 2021). Almost one-third of respondents endorsed facilitators around shorter measures and having more time to administer measures, in line with barriers identified from previous surveys highlighting the perceived time-consuming and burdensome nature of measures (Hatfield & Ogles, 2004; Ionita & Fitzpatrick, 2014; Ionita et al., 2020). The need for valid measures for specific populations has been seldom mentioned in the literature previously (Cho et al., 2021), and about a quarter of the sample in the present study endorsed this as a facilitator. As more practitioners practice MBC, gaps in available measures may become more apparent in terms of psychometrically valid, age-appropriate measures which have clinical cut-offs and normative data available for the clients’ population. Given the cultural landscape of Australia, measures that are culturally sensitive and appropriate for the diversity of MH consumers are also needed. These are key areas for further research.

Only around one in five practitioners endorsed additional training in EBA as a facilitator. Lack of training has been identified as a barrier in previous practitioner surveys (Ionita et al., 2020; Batty et al., 2013; Murphy et al., 2021). While our results suggest that CYMH practitioners in Australia endorse facilitators other than training more frequently, good quality training and support are necessary when using MBC (De Jong et al., 2023) to provide information about how to use measures, best interpret data, integrate feedback into sessions, and collaboratively adjust the course of treatment. Results comparing facilitators of measure use between MBC users and non-users showed users endorsed practical facilitators such as free measures, shorter measures, and better tools to administer measures significantly more than non-users. MBC users also endorsed a higher number of facilitators overall. These findings suggest that compared to non-users, MBC users may be more aware of key factors that would facilitate measure use and may hold more positive attitudes towards routine measure use, although further research would be needed to confirm this.

Regarding predictors of MBC use, none of the three predictors examined were found to be significantly associated with MBC use or non-use. Thus, there was no support for Hypothesis 1 that fewer years of clinical experience and not being in private practice would be associated with greater measure use, which is contrary to previous research (Jensen-Doss et al., 2018). Overall, this finding is encouraging as it suggests that these factors do not determine frequent measure use, and there may be opportunities to increase MBC use for all practitioners regardless of profession, context, and years of experience. However, it should be noted that the lack of significant predictors in this study may be due to the way that MBC was defined in this study, that is, to be inclusive of use at specific time points, as well as frequent use of measures. Restricting the MBC use grouping to include only frequent use of measures may have changed findings, but this was not possible due to low response numbers.

There are three implications to policy and practice regarding the use of measures and MBC. Firstly, there is a clear need to encourage evidence-based assessment in practice. At a minimum, measures should be implemented at assessment and discharge to aid assessment, treatment planning, discharge, and assist with program evaluation. However, pre-post use of measures does not help with progress monitoring and forecasting clients who may be at risk of non-response or drop out. Thus, there is a need to encourage ongoing use of measures as part of MBC. Secondly, some of the facilitators of measure use identified in this research are relatively easy to address, such as informing practitioners about the availability of free and brief measures of CYMH, such as the Pediatric Symptom Checklist-17 (Gardner et al., 1999). The PSC-17 is validated for children 4–17 years and has internalising, externalising and attention subscales. It is easy to use, simple to score and has excellent psychometrics (Becker-Haimes et al., 2020), and is ideally suited for use in MBC. Thirdly, even though training was only endorsed as a facilitator by a minority of practitioners, offering widespread training may be a way of disseminating information about the value of MBC and other facilitators, such as the availability of brief and free measures. Given the relative lack of research on MBC in CYMH, more research is needed on the efficacy of MBC as it may be that practitioners simply require more evidence about the positive effects to increase uptake. In fact, at present there is little research on the benefits of MBC for children under 12 years. Addressing this gap is a priority given attrition rates in CYMH interventions are high (Chacko et al., 2016) and effect sizes for intervention efficacy for child MH problems are not improving over time (Weisz et al., 2019). Future research should also explore what outcomes to measure and how frequently measures should be used, to better guide practitioners when using MBC.

The strengths of this study were a sample of practitioners, who were diverse in profession and workplace setting. However, there were two main limitations that need to be considered when interpreting results. Firstly, given the sample was not representative in terms of geographic location, there may have been sample biases which means findings are not generalisable to all Australian CYMH practitioners. Potential sample biases may have impacted the findings in various ways. For example, if psychologists were overrepresented in the sample, the rates of measure use may be higher given that psychologists are trained in evidence-based assessment. However, as there is no information available about the overall population of CYMH practitioners, it is difficult to determine representativeness of the sample based on profession or any other characteristics. It is important for future surveys to include larger samples and to attempt to obtain representativeness, although this is challenging when practitioners from multiple professional backgrounds are recruited. Secondly, as previously mentioned, MBC involves frequent measure use along with feedback provided to clients to inform collaborative discussions about treatment planning. The feedback component is critical, but was not asked about in this study, so future surveys should ensure that questions comprise all components of MBC.

This survey of Australian practitioners working in CYMH examined the use of measures in clinical practice and found that most practitioners reported using measures at some stage, but only a minority used measures frequently. The survey identified several facilitators of measure use which are easy to implement and may increase the use of measures in the Australian context. It was encouraging to find that factors such as years of experience, profession, and context do not appear to be associated with measure use, as there is a clear need to encourage practitioners to adopt evidence-based assessment and MBC in their clinical work, in an effort to improve client engagement and outcomes, and ultimately strengthen the CYMH system.

## Data Availability

The data that support the findings of this study are available on request from the corresponding author, LT. The data are not publicly available due to their containing information that could compromise the privacy of research participants.
